# A novel assessment of abdominal pseudohernia after thoracolumbar vertebral compression fractures using surface electromyography and ultrasonography: A case report

**DOI:** 10.1097/MD.0000000000024973

**Published:** 2021-03-05

**Authors:** Min Soo Choi, Myung Hun Jang, Byeong Ju Lee, Yong Beom Shin, Sang Hun Kim

**Affiliations:** a Department of Rehabilitation Medicine, Biomedical Research Institute, Pusan National University Hospital; b Department of Rehabilitation Medicine, Biomedical Research Institute, Pusan National University Hospital and Pusan National University School of Medicine, Busan, Republic of Korea.

**Keywords:** abdominal hernias, electromyography, radiculopathy, ultrasonography

## Abstract

**Rationale:**

An abdominal pseudohernia is a protrusion of the abdominal wall that there is no actual muscular disruption. This report presents a case in which abdominal muscle activities were accurately and quantitatively measured using ultrasonography (US) and surface electromyography in a patient with abdominal pseudohernia.

**Patient concerns:**

A 62-year-old man presented with a marked protrusion on the left abdomen with increasing abdominal pressure.

**Diagnoses:**

First, the thickness of the abdominal muscle was measured with US while the patient constantly blew the positive expiratory pressure device. When the force was applied to the abdomen, the mean thickness of the muscle layer on the lesion site was found to be thinner. Second, the activities of the abdominal muscles were measured using surface electromyography by attaching electrodes to 8 channels at the same time. When the same pressure was applied on both sides of the abdomen, more recruitment occurred to compensate for muscle weakness at the lesion site. Through the previous 2 tests, the decrease in muscle activity in the lesion area could be quantitatively evaluated. Third, the denervation of the muscle was confirmed using US-guided needle electromyography.

**Interventions:**

The patient in this case was wearing an abdominal binder. In addition, he had been training his abdominal muscles through McGill exercise and breathing exercises such as with a positive expiratory pressure device.

**Outcomes:**

The patient was able to understand his symptoms. A follow-up test will be performed to see if there is any improvement.

**Lessons:**

By using these outstanding assessment methods, proper diagnosis and rehabilitation treatment strategies can be developed.

## 1 Introduction

An abdominal pseudohernia is a protrusion of the abdominal wall that resembles a hernia but differs from a true hernia in that it has no actual muscular disruption and all muscle and fascial layers remain intact.^[1]^ Pseudohernia is a rare phenomenon, which has been reported in association with various syndromes involving peripheral neuropathy, including zoster infection,^[2]^ diabetes mellitus,^[3,4]^ and following operative trauma.^[5–7]^

Until now, pseudohernia has often been neglected, and cases are rarely reported. However, pseudohernia can cause anxiety in patients, cosmetic discomfort in daily life, and cause various complications such as gastrointestinal problems or gait disturbance. According to 1 report, 19.4% of patients with abdominal pseudohernia complain of gastrointestinal complications, and the most common complication was constipation.^[2]^ Other reports have described cases in which abdominal pseudohernia triggered scoliosis, and standing and gait disturbances.^[8]^ Therefore, the cause of pseudohernia must be identified and the degree of nerve damage and muscle weakness should be quantitatively examined.

Conventionally, electromyography (EMG), computed tomography (CT), and magnetic resonance imaging (MRI), have been introduced as methods of diagnosing abdominal pseudohernia.^[9]^ As CT and MRI are static tests, analysis of muscle activity has limitations. This report presents a case in which abdominal muscle activities were accurately and quantitatively measured using and ultrasonography (US) and surface electromyography (sEMG) in a patient with abdominal pseudohernia.

## 2 Case presentation

A 62-year-old man fell from a height of 4 m on March 26, 2020. He did not have any history of neurological, psychological, or genetic disorders. He complained of severe low back pain. When he visited the emergency department, he did not complain of muscle weakness or decreased sensation in both lower extremities. Physical examination confirmed no neurological deficit caused by the spinal cord injury. Recent compression fractures at T11, 12, and L1 and narrowing of the left T11 neural foramina were observed in the T-L junction on MRI. Therefore, surgery for posterior fusion of T10–L2 was performed on March 31. The patient's general condition improved, and he was transferred to the rehabilitation department on April 14 for active rehabilitation treatment.

**Figure 1:**
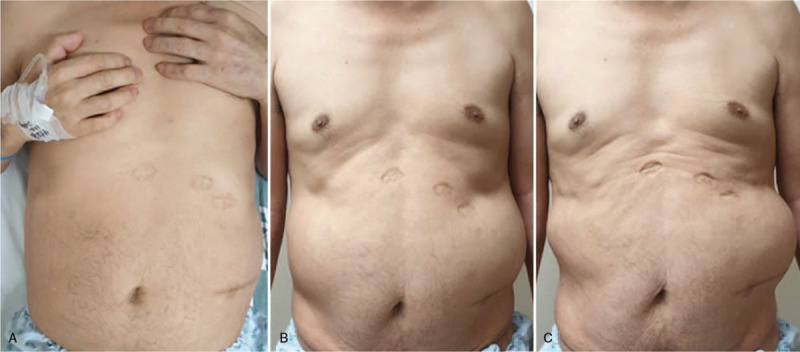
Approximately 15 × 15 cm marked protrusion on the left side of abdomen in the area innervated by the 11th thoracic nerve when the supine (A), standing (B) position, and Valsalva maneuver were performed (C).

With increasing abdominal pressure, for example, when standing up or coughing, an approximately 15- × 15-cm marked protrusion on the left flank following the T11 dermatome was observed (Fig. 1). No palpable mass, pain, tenderness, or rebound tenderness was observed. Previously, he was in a bedridden state, so he did not notice such symptoms. The patient complained of hypoesthesia confined to the left T11 dermatome, and some gastrointestinal symptoms such as abdominal discomfort and dyspepsia.

Abdominal hernia was ruled out because no defects were found in the musculofascial layers of the abdominal wall in the initial abdominal CT scan. In addition, hematoma and ascites were not detected in the CT scan. Therefore, the cause of the abdominal protrusion was considered to be a pseudohernia. To confirm this, 3 tests were performed. To quantitatively analyze muscle activity, US and sEMG were used to demonstrate muscle weakness and needle EMG to demonstrate denervation.

**Figure 2:**
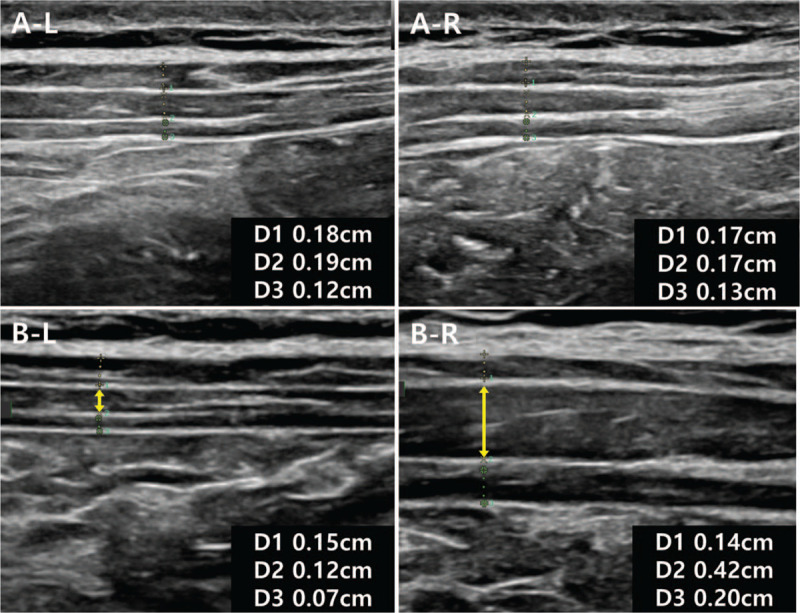
The thicknesses of the 3 abdominal muscle layers, namely the external oblique (D1), internal oblique (D2), and transversus abdominis muscles (D3), were measured using ultrasonography in the supine (A) and standing positions while the patient blew the positive expiratory pressure device constantly at 20% strength of the measured maximal expiratory pressure (B). Each measurement was performed in the area with the most severe herniation (left, L) and on the opposite side (right, R).

First, the thicknesses of the 3 layers of abdominal muscles were measured using US (Fig. 2). Measurements were performed in the area with the most severe herniation and on the opposite side. A 2-step test was conducted as follows: first, while the patient was resting in the supine position and, second, while the patient blew the positive expiratory pressure (PEP) device (Threshold PEP, Philips, Monroeville, PA) constantly at 20% strength of the measured maximal expiratory pressure (MEP) in the standing position. The MEP was assessed in a standardized manner using a desktop spirometer (Pony FX, Cosmed, Rome, Italy) to assess the strength of the respiratory muscles. Each stage was repeated 3 times, and the average value was obtained. The thicknesses of the 3 muscle layers, namely the external oblique (EO), internal oblique (IO), and transverse abdominis (TrA), were measured.

**Figure 3:**
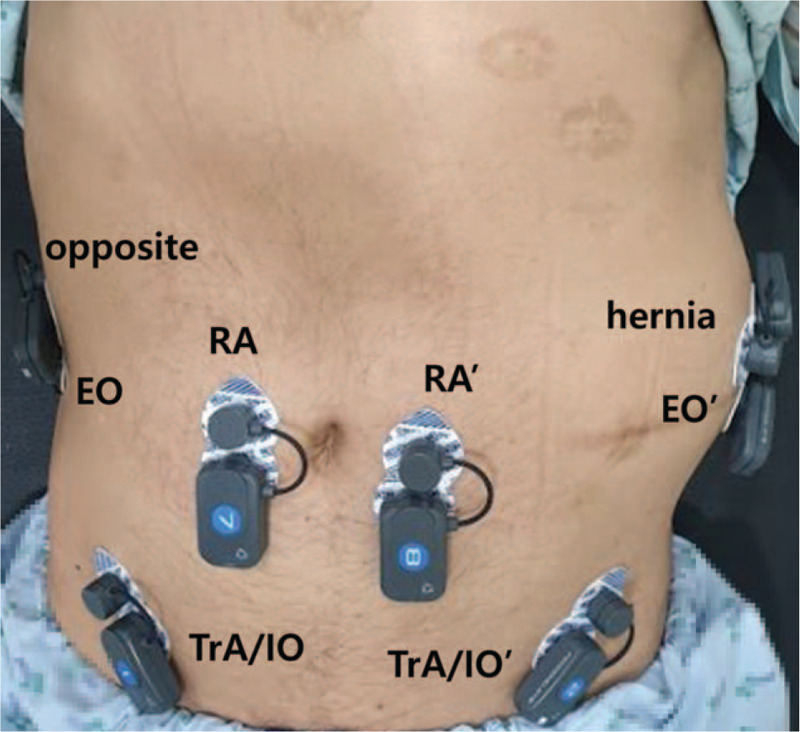
Measuring the activity of the muscles by attaching electrodes to a total of 8 locations, both rectus abdominis (RA), both external oblique (EO), internal oblique/transversus abdominis (IO/TrA) muscles, and the most severe herniation area and the opposite side.

Second, the activities of the abdominal muscles were measured using sEMG by attaching electrodes to 8 channels at the same time, on both rectus abdominis (RA), both EO, both IO/TrA muscles, and the most severe herniation area and opposite side. Conductive adhesive hydrogel snap electrodes (Covidien 533 foam electrodes, Covidien: 710 Medtronic Parkway, Minneapolis, MN, USA) were used for sEMG. The electrode was composed of a 1-cm-sized circular conductive area. The electrodes were placed parallel to the muscle fiber orientation, and the distance between the centers of each electrode was 2 cm. The electrode recording RA muscle activity was attached 2 cm lateral to the umbilicus. The electrode recording the EO muscle activity was attached laterally to the RA and directly above the anterior superior iliac crest, halfway between the crest and the ribs at a slightly oblique angle, and the electrode recording the IO/TrA muscle activity was attached 2 cm medially and below the anterior superior iliac spine (Fig. 3).^[10,11]^ Muscle activity was measured using the BTS-FREEEMG 1000 device (BTS Bioengineering, Milan, Italy) with a sampling frequency of 1 kHz, and the band-pass was filtered at 20 to 500 Hz.

The activities of the muscles while the patient blew the PEP device at 20% intensity of the MEP were compared. The percentage of the reference voluntary contraction (% RVC) was calculated for normalization by using the muscle activity during the blowing at the MEP. The % RVC of each muscle was determined using the following equation:

Muscle activation intensity (%)=(mean RMS of 20% MEP)/(RMS of the MEP)×100

The root mean square (RMS) of each muscle was analyzed at 3 seconds in the middle of the 5-second blowing. After 3 repeated measurements, the maximum RMS value within 20% of the mean RMS value was selected. This process was conducted in a standing position, with a break of at least 3 minutes between each measurement. The results are shown in Table 1.

**Table 1:** Measured root mean square and % reference voluntary contraction values of the abdominal muscles using surface electromyography.

Electrode	MEP	20% MEP		
channel	RMS (uV)	RMS (uV)	% RVC^∗^	R/L ratio
EO				
Right	25.8	10.8	41.9	1.62
Left	19.8	13.4	67.7	
RA				
Right	11.7	3.6	30.8	1.78
Left	7.5	4.1	54.7	
Hernia				
Right	40.6	22.5	55.4	1.49
Left	17.4	14.4	82.8	

Third, a US-guided needle EMG was performed to confirm the presence of denervation potential. Under US guidance, the EMG needle was inserted in the bilateral IO muscle, and the insertional activity, resting potential, and motor unit potential during volition were measured.

The patient wore an elastic binder during activities to prevent protrusion of the abdominal wall. In addition, he had been training his abdominal muscles through McGill exercise and breathing exercises using abdominal muscles, such as with a PEP device.

After the study, the patient was discharged. It was supposed to check the progress of recovery in out-patient department, but the patient did not come for personal reasons.

### 2.1 Ethics statement

As it is a case report with written informed consent, our institutional review board does not require formal ethical approval. Written consent was obtained from the patient for publication of this case report.

## 3 Discussion

On the basis of the abdominal US results, when force was applied to the abdomen, the thickness of the right abdominal muscle layers became thicker than in the resting state, and the IO muscle was the most contracted. On the other hand, in the thickness of the abdominal muscle in the left lesion site became rather thinner. The muscles in the lesion area, where the contraction power of the muscle was weak, was thought to have stretched as the abdominal pressure increased.

The sEMG signals were found in both abdominal muscles, and the % RVC value of the muscle activity of the left abdominal muscle was greater than that of the right abdominal muscle. This may be because the left abdominal muscle activity increased to maintain muscle strength under the same load.

As the electrode of the sEMG has a wide recording field, cross-talking exists. Therefore, a needle EMG was performed to confirm the actual motor unit action potential. Whereas the EMG signal of the right IO muscle was normal, the EMG signal of the left IO muscle indicated increased insertional activities and abnormal spontaneous activities of positive sharp waves and fibrillations at rest. In addition, decreased recruitment of motor unit action potential was observed on volition.

Conventionally, CT and needle EMG have been introduced as methods of diagnosing abdominal pseudohernia. CT should be used to rule out the possibility of abdominal wall hernia due to structural defect, mass, or fluid collection. Needle EMG can be used to detect nerve conduction abnormality in the abdominal wall muscles.^[9]^ We additionally used US and sEMG to dynamically observe the movement of abdominal muscles. In addition, the reproducibility of the test was improved by applying a certain pressure to the abdomen using a PEP device. However, on repeated tests, the same pressure was not always applied to the abdomen. To compensate for this, we calculated the ratio of the values in the normal and lesion-side areas to indicate the degree of weakness.

In this case, data on the progress of recovery cannot be obtained due to follow up loss. In the future, we plan to conduct research on the effectiveness of interventions using these tools serially.

This report presents simple, objective, and quantitative tests of pseudohernia. By using these outstanding assessment methods, proper diagnosis and rehabilitation treatment strategies can be developed. Furthermore, evaluating whether the symptoms have improved through follow-up tests is expected to have a positive effect on the improvement of the patient's quality of life. In further studies, it will be necessary to develop a more standardized evaluation protocol.

This study conforms to all CARE guidelines and reports the required information accordingly.

## Author contributions

**Conceptualization:** Sang Hun Kim, Byeong Ju Lee.

**Data curation:** Min Soo Choi, Myung Hun Jang.

**Investigation:** Min Soo Choi, Myung Hun Jang.

**Methodology:** Sang Hun Kim, Myung Hun Jang.

**Supervision:** Sang Hun Kim, Yong Beom Shin.

**Validation:** Min Soo Choi, Byeong Ju Lee.

**Visualization:** Min Soo Choi.

**Writing – original draft:** Min Soo Choi.

**Writing – review & editing:** Sang Hun Kim, Yong Beom Shin.

## References

[1] Butensky AM Gruss LP Gleit ZL. Flank pseudohernia following posterior rib fracture: a case report. J Med Case Rep 2016;10:273. 27716425 10.1186/s13256-016-1054-9 PMC5045639

[2] Chernev I Dado D. Segmental zoster abdominal paresis (zoster pseudohernia): a review of the literature. PM R 2013;5:786–90. 24054853 10.1016/j.pmrj.2013.05.013

[3] Weeks R Thomas P Gale A. Abdominal pseudohernia caused by diabetic truncal radiculoneuropathy. J Neurol Neurosurg Psychiatry 1999;66:405–1405. 10084546 10.1136/jnnp.66.3.405 PMC1736241

[4] Kesler A Galili-Mosberg R Gadoth N. Acquired neurogenic abdominal wall weakness simulating abdominal hernia. Isr Med Assoc J 2002;4:262–4. 12001699

[5] Gardner GP Josephs LG Rosca M. The retroperitoneal incision: an evaluation of postoperative flank ’bulge’. Arch Surg 1994;129:753–6. 8024457 10.1001/archsurg.1994.01420310085015

[6] Chatterjee S Nam R Fleshner N Klotz L. Permanent flank bulge is a consequence of flank incision for radical nephrectomy in one half of patients. Paper Presented at: Urologic Oncology: Seminars and Original Investigations; 2004. 10.1016/S1078-1439(03)00099-1 14969802

[7] Durham-Hall A Wallis S Butt I. Abdominal wall pseudohernia following video-assisted thoracoscopy and pleural biopsy. Hernia 2009;13:93–5. 18584279 10.1007/s10029-008-0401-1

[8] Tashiro S Akaboshi K Kobayashi Y. Herpes zoster–induced trunk muscle paresis presenting with abdominal wall pseudohernia, scoliosis, and gait disturbance and its rehabilitation: a case report. Arch Phys Med Rehabil 2010;91:321–5. 20159140 10.1016/j.apmr.2009.10.011

[9] Chernev Ivan DADO David. Segmental zoster abdominal paresis (zoster pseudohernia): a review of the literature. PM R 2013;5:786–90. 24054853 10.1016/j.pmrj.2013.05.013

[10] Montes AM Baptista J Crasto C. Abdominal muscle activity during breathing with and without inspiratory and expiratory loads in healthy subjects. J Electromyogr Kinesiol 2016;30:143–50. 27434376 10.1016/j.jelekin.2016.07.002

[11] Marshall P Murphy B. The validity and reliability of surface EMG to assess the neuromuscular response of the abdominal muscles to rapid limb movement. J Electromyogr Kinesiol 2003;13:477–89. 12932422 10.1016/s1050-6411(03)00027-0

